# Does intraocular straylight predict night driving visual performance? Correlations between straylight levels and contrast sensitivity, halo size, and hazard recognition distance with and without glare

**DOI:** 10.3389/fnhum.2022.910620

**Published:** 2022-09-13

**Authors:** Judith Ungewiss, Ulrich Schiefer, Peter Eichinger, Michael Wörner, David P. Crabb, Pete R. Jones

**Affiliations:** ^1^Competence Center “Vision Research”, Study Course Ophthalmic Optics and Optometry, University of Applied Sciences, Aalen, Germany; ^2^Carl Zeiss Vision International GmbH, Aalen, Germany; ^3^Department of Ophthalmology, University of Tübingen, Tübingen, Germany; ^4^Study Course Mechatronics, University of Applied Sciences, Aalen, Germany; ^5^Blickshift GmbH, Stuttgart, Germany; ^6^Department of Optometry and Visual Sciences, School of Health Sciences, University of London, London, United Kingdom

**Keywords:** driving simulator, contrast sensitivity, straylight, halo, glare, nighttime driving, hazard, recognition distance

## Abstract

**Purpose:**

To evaluate the relationship between intraocular straylight perception and: (i) contrast sensitivity (CS), (ii) halo size, and (iii) hazard recognition distance, in the presence and absence of glare.

**Subjects and methods:**

Participants were 15 (5 female) ophthalmologically healthy adults, aged 54.6–80.6 (median: 67.2) years. Intraocular straylight (log s) was measured using a straylight meter (C-Quant; Oculus GmbH, Wetzlar, Germany). CS with glare was measured clinically using the Optovist I device (Vistec Inc., Olching, Germany) and also within a driving simulator using Landolt Cs. These were presented under both *static* or *dynamic* viewing conditions, and either *with* or *without* glare. Hazard detection distance was measured for simulated obstacles of varying contrast. For this, the participant was required to maintain a speed of 60 km/h within a custom-built nighttime driving simulator. Glare was simulated by LED arrays, moved by cable robots to mimic an oncoming car’s headlights. Halo size (“halometry”) was measured by moving Landolt Cs outward originating from the center of a static glare source. The outcome measure from “halometry” was the radius of the halo (angular extent, in degrees visual angle).

**Results:**

The correlation between intraocular straylight perception, log s, and hazard recognition distance under glare was poor for the low contrast obstacles (leading/subdominant eye: *r* = 0.27/*r* = 0.34). Conversely, log CS measured with glare strongly predicted hazard recognition distances under glare. This was true both when log CS was measured using a clinical device (Optovist I: *r* = 0.93) and within the driving simulator, under *static* (*r* = 0.69) and *dynamic* (*r* = 0.61) conditions, and also with “halometry” (*r* = 0.70). Glare reduced log CS and hazard recognition distance for almost all visual function parameters.

**Conclusion:**

Intraocular straylight was a poor predictor of visual function and driving performance within this experiment. Conversely, CS was a strong predictor of both hazard recognition and halo extent. The presence of glare and motion lead to a degradation of CS in a driving simulator. Future studies are necessary to evaluate the effectiveness of all above-mentioned vision-related parameters for predicting fitness to drive under real-life conditions.

## Introduction

Vision is our most valued sense (Enoch et al., 2019) and is, by some estimates, responsible for encoding over 80% of all sensory information (Taylor, 1982). Contrast sensitivity (CS) in particular seems to be important for safe driving – outweighing spatial resolution (i.e., visual acuity, VA) in terms of its ability to predict nighttime recognition performance in motorists (Wood and Owens, 2005).

Human vision is especially challenged under nighttime (mesopic) conditions, where CS is reduced profoundly compared to daytime (photopic) illumination (Hertenstein et al., 2016). This is likely one reason why the risk of a fatal car accidents increases by 50% at night compared to daylight conditions (Kraftfahrtbundesamt, 2020; Statistisches Bundesamt, 2020). However, in addition to illumination conditions, other factors such as sleepiness or the consumption of alcohol by drivers are also likely to have an impact on this. Our aging societies therefore pose a worrying challenge to safe night driving. For instance, within the EU alone, the proportion of people aged 65 years and older is expected to increase from 19.3% (in 2020) to 27.3% (in 2050) (Bundeszentrale für politische Bildung, 2018). While fatalities involving drivers over 65 years of age have increased continuously over the past decade, and are expected to further raise over the upcoming years (Hu, 2001), the age-related rate of crash involvement, corrected for yearly mileage, shows a u-shaped course. High rates occur below the age of 25 years (mostly due to inexperience and/or drug/alcohol abuse) and increasing rates can be seen beyond the age of 70 years (Cerrelli, 1998).

There is an increasing awareness of the particular challenges posed by nighttime driving. Recently 64% of motorists reported at least one hazardous traffic incident due to glare and 80% of motorists expressed the desire for vision improvement during night-time driving. Indeed, more than 60% stated that they would spend at least 300 € for considerably better vision when driving at night (Zydek, 2014).

Disability glare is defined as glare that impairs the visibility of objects (International Commission on Illumination [CIE], 2020). It can be understood in terms of light scatter within the eye, with intense light sources “veiling” the contrast of other objects within the visual scene (Bullough, 2017). In the course of the last decades, glare exposure for road users has increased due to the installation of halogen and LED headlamps with higher luminance levels in vehicles.

There are analytic formulae for the prediction and description of disability glare available. Vos describes several formulae indicating a relationship between age and disability glare (Vos, 2003). It may seem questionable whether the influencing factor for glare is not really age – but rather the opacity of the optical media. Age is related to changes in the opacity of the optical media and thus to changes of susceptibility to glare for the average of a population. However, individuals can show exceptional cases, for instance of juvenile cataract. In these cases, formulae based on age alone do not seem to be sufficient for individual diagnostics.

Furthermore, various studies have attempted to quantify empirically the associations between changes in vision, glare perception, and driving ability. For example, Finger et al. (2011) evaluated visual function and Quality of Life (QoL) under low luminous conditions using the German low luminance questionnaire. They found that low luminance functioning, as reported by participants via the German low luminance questionnaire, was considerably compromised even in cases of mild photopic VA loss (Finger et al., 2011). This indicates that even mild reductions of photopic vision parameters may lead to substantial impairment under mesopic conditions.

In terms of research into straylight specifically, van den Berg et al. (2013) used the C-Quant device and found that straylight contributes to the quality of vision and could also be used as an issuing criterion for driving licenses. Tuan et al. (2006) found that wavefront analysis systems were capable of predicting visual symptoms and complaints amongst patients with high-order aberrations following laser eye surgery.

In terms of studies specifically examining driving ability, Spreng et al. (2018) assessed the degree to which age related changes in CS are associated with VA, driving restrictions, and daytime on-road driving evaluations in elderly drivers (over 70 years old). They found that a loss of CS was common in elderly drivers. Wood and Owens (2005) examined whether VA and CS, both measured under a range of luminance conditions, were able to predict drivers’ on-road recognition performance, but they found that standard, photopic measures of VA were not capable of predicting drivers’ recognition performance under both, day- and nighttime conditions (Wood and Owens, 2005).

Drivers tend to adjust their speed more precisely under low-contrast than under clear view conditions, which means that they tend to drive slower under low-contrast conditions [as reported by Owens et al. (2010) using diffusion filters during daytime driving – though note that this may not necessarily mimic the age-related changes in CS observed in older drivers under scotopic conditions]. Visual recognition is degraded during nighttime driving, especially for older drivers. And while drivers especially for advanced ages tend to drive slower under low light conditions, these speed reductions are believed to be insufficient to compensate for the age-related decrease in visibility (Owens et al., 1999).

Finally, von Hebenstreit (1984) showed that the frequency of accidents was significantly greater amongst people with reduced VA, while Owsley et al. (2001) found the same results for reduced CS due to cataract – even if only one eye was affected.

Currently, in addition to photopic testing, CS assessments under mesopic conditions are increasingly being used to assess twilight vision as part of driving aptitude examinations. However, due in part to the logistical difficulties involved in running real-world night-driving assessments, it remains unclear whether and to what extent mesopic CS measures are capable of accurately predicting night driving ability. Cutting-edge driving simulators may offer a solution. They allow for the standardized presentation of numerous realistic driving situations – both, under photopic and under night driving conditions. To date, however, it has been difficult to simulate the all-important effect of headlight glare from oncoming vehicles. In the present project, innovative approaches, deviating from those of the Fraunhofer Institute for Industrial Engineering (IAO) (Melcher et al., 2011) and of Harvard Medical School (Hwang and Peli, 2013; Hwang et al., 2018), are introduced and adapted for various driving simulator environments: LED light arrays, moved by cable robots, are used to realistically simulate glare from oncoming car headlights.

The purpose of this study was to evaluate the relationship between intraocular straylight perception and: (i) CS, (ii) halo size, and (iii) hazard recognition distance (HRD), with and without the presence of glare.

## Materials and methods

### Participants

Inclusion criteria were: age ≥ 18.0 years; refractive media clear or at maximum moderate lens opacity at slit lamp examination; maximum spherical ametropia ±5 dpt, maximum cylindrical ametropia 2.5 dpt; normal 90° visual field as assessed using the Octopus 900 perimeter (program FG “driver’s license,” grid 105 locations, eccentricity 0° to 80°, strategy “2-LT,” stimulus size III, stimulus duration 200 ms, background luminance 10 cd/m^2^, phase 1; Haag-Streit, Köniz, Switzerland).

Participants who reported disorders of the visual pathways, severe ophthalmic injuries or inflammations, ocular motility disorders and/or double vision and status post ophthalmic surgery (including cataract surgery with intraocular lens implantation) were excluded. Furthermore, a binocular distant VA (habitual correction) under photopic conditions of less than 0.5 (corresponding to a Snellen fraction of 10/20 or to log VA 0.3) led to an exclusion of the respective participant.

Participants were recruited by circular emails within the Aalen University of Applied Sciences, Aalen, Germany, by newspaper advertisements, and via ophthalmologists in private practice.

The final contact of the Vision Research Center at Aalen to the tested subjects was made by telephone, exclusively by the clinical investigator US.

Written informed consent was obtained from each tested subject. The study followed the tenets of the Helsinki declaration and subsequent updates. The study protocol and its amended version were approved by the Institutional Review Board of the State Medical Association of Baden-Württemberg (F-2015-044#A2). The original study protocol was approved by ClinicalTrials.gov (NCT03169855; last update 08-01-2019).

Only persons with complete vaccination protection and a negative antigen test for COVID-19 on the day of the experiment were admitted to the experiments.

### Methods

Participants were examined according to the following procedure: clinical examinations (see section “Clinical examinations”), examination of CS in the driving simulator (see section “Examination of contrast sensitivity in the driving simulator”), examination of the HRD in the driving simulator (see section “Examination of the hazard recognition distance in the driving simulator”), and halometry examination (see section “Halometry examination”).

**FIGURE 1:**
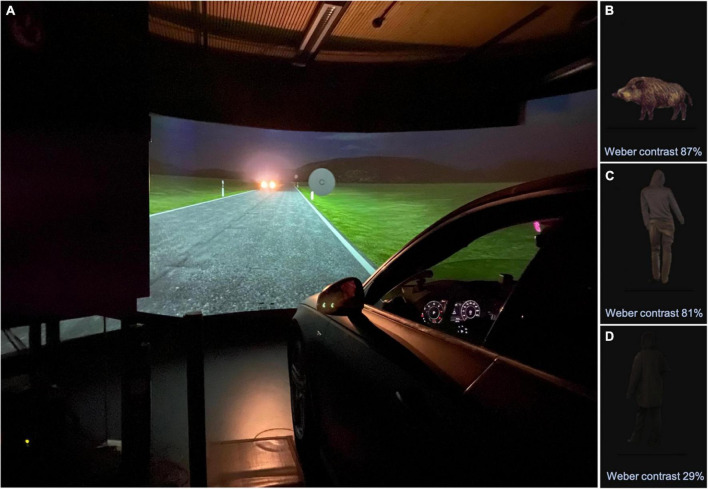
**(A)** The fixed-base night driving simulator at the Aalen Mobility Perception and Exploration Lab (AMPEL, Competence Center “Vision Research”), located at the Innovation Centre at the University of Applied Sciences, Aalen/FRG. Visible on the right is the modified simulator vehicle (Audi A4, Audi AG, Ingolstadt/FRG), with fully digital dashboard and implemented, contactless eye and head tracking device (SmartEye, Gothenburg/SE). Visible on the left is the housing of one of the two high-performance LED planetarium projectors (Zeiss AG, Jena/FRG) - the housing provides sound insulation and cooling. In the center of the image can be seen the cylindric projection screen (radius: 3.2 m) showing a night-time driving scenario with an oncoming vehicle on a rural road with dipped headlight. These oncoming headlights were simulated by two LED arrays, moved by cable robots precisely along the trajectories required to mimic an oncoming car. WLAN signal transmission was further used precisely adjust the luminance and angle of the LED panels in a realistic fashion. As shown here, optotypes of varying visual acuity and contrast sensitivity (CS) levels, as well as obstacles of varying CS levels (not shown) were presented directly to the right of the roadside. This were presented against a uniform gray circular background. The three obstacles are shown in panels **(B–D)**, as follows. **(B)** “Wild boar” (Weber contrast 87%), **(C)** “man dressed in gray” (Weber contrast 81%), **(D)** “man dressed in black” (Weber contrast 29%).

Experiments were conducted in the nighttime driving simulator of the Aalen Mobility Perception and Exploration Lab (AMPEL) – for more information, see Figure 1A. Two calibrated LED arrays with filter attachment (filter foil Lee 204 Full C.T. orange, manufacturer Lee Filters, Hampshire, United Kingdom), moved by cable robots, simulated *static* as well as *dynamic glare* conditions, corresponding to the trajectories, viewing angle (between −20° and −7°), and luminance characteristics (illuminance between 0.04 and 1.35 lux) of the (low beam) headlights of an approaching Golf/Rabbit VII (Volkswagen AG, Wolfsburg, Germany). To ensure that the simulator correctly simulated real-world headlight glare, spot luminance calibration procedures were performed prior to the start of the study, using the Spectroradiometer CAS 140 VIS/UV (Instrument Systems GmbH, Munich, Germany) and the Minolta Luminance Meter LS160 (Konica Minolta Holdings K.K., Tokyo, Japan). For measurements of the spatial distribution of luminance of the simulated night scene in the absence of glare (as a baseline for headlight glare simulation), a digital camera Canon EOS 600D (Canon Inc., Tokyo, Japan) was applied, calibrated via comparison with the Spectroradiometer CAS 140 VIS/UV. Depending on the (virtual) distance (range 10–40 m), the apron luminance within the simulator setting varied between 1.3 and 2 cd/m^2^ for the right and between approximately 0.6 and 1 cd/m^2^ for the left roadside, respectively. The luminance characteristics of the simulator setting corresponded (maximum deviation: 20%) to the on-road conditions of a vehicle with dimmed halogen headlights (Audi A4, Audi AG, Ingolstadt, Germany). For more information on the driving simulator (see Ungewiss et al., 2020, 2022; Schiefer et al., 2021) and Supplementary material (additional information on the calibration of the headlamps).

De-identified data were analyzed and visualized using the statistical software JMP 16 PRO (SAS Institute GmbH, Heidelberg, Germany) as well as R (Version 4.1.0, GUI 1.76, R Foundation for Statistical Computing, 2021).

#### Clinical examinations

Ophthalmic and general history taking, as well as a detailed ophthalmologic examination, had been performed in a previous phase of this study. This included the assessment of ocular alignment (cover test) and ocular motility (evaluation of smooth pursuit and saccadic velocity in horizontal and vertical direction), examination of the anterior and middle eye segments with a slit lamp (BQ 900, LED powered, Haag-Streit, Köniz, Switzerland). High contrast (Weber contrast 100%) and low contrast (Weber contrast 5%) VA thresholds (adaptive BestPEST strategy) under photopic conditions were assessed by the Freiburg Visual Acuity and Contrast Test (FrACT; see Bach, 1996), with 22 presentations of eight-position Landolt Cs using an adaptive BestPEST thresholding procedure (see Treutwein, 1995; Bach, 2006). For the transformation of CS levels, see Table 1 and/or Bach et al. (2017). CS under *clinical conditions*, i.e., the twilight (mesopic) vision and glare sensitivity, each under binocular conditions, was assessed by a modified Optovist I instrument, Vistec AG, Olching, Germany) that allowed the presentation of low contrast levels (down to a Weber contrast of 9%). During this procedure, eight-position Landolt Cs (VA level 0.1, corresponding to logMAR 1.0) were presented with varying contrast levels *without* (background luminance 0.032 cd/m^2^) and *with* glare (glare source: visual angle: 0.25°, eccentricity 3° to the left, corneal illumination level 0.35 lux, background luminance 0.1 cd/m^2^), using a three out of five criterion. Intraocular straylight perception, log (s), under monocular conditions, was measured by the C-Quant device (“straylight meter,” Oculus, Wetzlar, Germany).

**TABLE 1:** Relation between different contrast definitions (see Bach et al., 2017).

Stimulus	log CS	Weber contrast (%)	Michelson contrast (%)	Aulhorn contrast	Technical contrast
Wild boar	0.06	87	77	1:7.8	7.8:1
Gray man	0.09	81	69	1:5.3	5.3:1
Black man	0.54	29	17	1:1.4	1.4:1
	0.50	32	19	1:1.46	1.46:1
	1.00	10	5	1:1.11	1.11:1
	1.50	3	2	1:1.03	1.03:1

#### Examination of contrast sensitivity in the driving simulator

Within the *driving simulator* (Figure 1) CS thresholds were assessed by presenting eight-position Landolt Cs (VA level 0.1, corresponding to logMAR 1.0) with varying contrast levels comparable to the above-mentioned clinical test procedure, using an adaptive BestPEST strategy under *static* and *dynamic* conditions, each *with* and *without* glare. Additionally, obstacles with three different contrast levels were presented under the four above-mentioned conditions: A “wild boar” (Weber contrast 87%), a “gray man” (Weber contrast 81%), and a “black man” (Weber contrast 29%), respectively (see Figures 1B–D).

For object contrast values, the average luminance values of several areas of each object have been measured (e.g., left shoulder, right shoulder, belly, head, etc.). An unweighted average of luminance values of these areas is considered as an indication for the contrast level of this object type.

Table 1 shows the relation between different contrast definitions according to Bach et al. (2017) highlighting in particular the values relevant for the present study. In section “Results”, the corresponding measured CS thresholds, log CS, are given. CS is calculated as reciprocal value of the respective Weber contrast. However, for readers more familiar with other contrast metrics, these log CS values can be converted to other contrast expressions using Table 1.

In the case of the static conditions (with and without glare), the participant’s vehicle was virtually parked at a distance of 50 m on the opposite lane, and remained stationary. This was simulated by having the two LED arrays adjusted with respect to the visual angle and headlight illuminance levels and remain static at this position during the measurements.

In the case of *dynamic* conditions (*with* and *without glare*), the participant first accelerated the simulated vehicle to a (virtual) speed of 90 km/h, covered a distance of approximately 550 m at this speed (which should be kept as constant as possible; this related to time span of on average 22 s for re-adaptation after being exposed to glare), and then – following a corresponding speed limit sign – reduced the speed to 60 km/h (and were again asked to maintain this speed as constantly as possible). In addition to the speedometer (with digital and pointer display), the virtual engine noise played into the passenger compartment served as an additional acoustic feedback signal for speed control. After a distance of approximately 300 m the aforementioned Landolt Cs (VA level 0.1, corresponding to logMAR 1.0) were presented at different contrast levels, by means of an adaptive threshold-determining algorithm (*see above*). CS was determined in a run of 22 trials.

For all examinations in the driving simulator, scenarios *without glare* preceded those *with glare*. Thus, a practice effect may have occurred.

#### Examination of the hazard recognition distance (HRD) in the driving simulator

Under dynamic conditions, the detection distance (remaining distance to the hazard when detected, i.e., when the subject stated “now”) and the corresponding HRD (remaining distance of the hazard when recognized, i.e., when the correct verbal specification of the aforementioned three obstacle types: “wild boar,” “man dressed in gray,” and “man dressed in black”) were additionally determined. Immediately following the subject’s response, the obstacles were blanked out. HRD was reported as *positive* if the subject responded *before* passing the obstacle. Subjects’ responses *after* the passage obstacle were deemed as *negative*. In almost all cases, the verbal specification occurred immediately after the “now” response (i.e., “in one go”), insofar it was assumed that the difference between the recorded detection and recognition distance was mainly attributable to the time span required to verbalize the response. Therefore, the detection distance was taken as the most immediate measure of the perception of the obstacle. Each of the three aforementioned conditions was presented three times, in randomized sequence, and the median of the corresponding distances was determined as a continuous outcome variable – thus, a total of nine presentations were made within the scope of this test section.

#### Halometry examination

**FIGURE 2:**
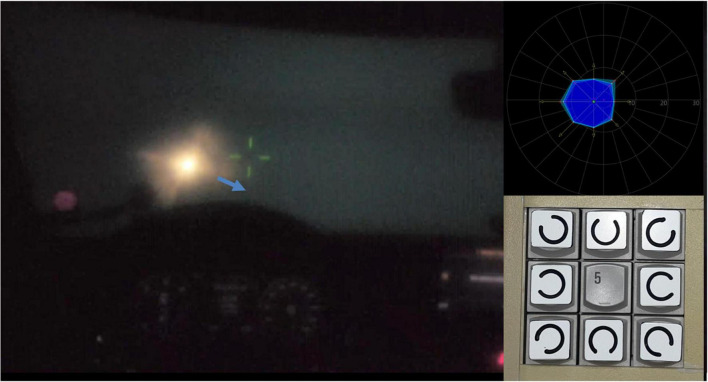
Illustration of the basic principle of “halometry”; patient’s view with a simulated cataract: a Landolt C, originating from the center of the glare source is crossing the border of the halo area (the origin of the blue arrow indicates the gap position of the optotype and the arrowhead shows the movement direction of the optotype). *Right upper insert*: examination result for the eight cardinal vectors demarcating the extent (eccentricity) of the halo, shaded in deep blue; the light blue area indicates the intra-subjects scatter, measured by repeated presentation of optotypes along each vector. *Right lower insert*: modified keypad (Michael Bach, University Eye Hospital, Freiburg, Germany) for the manual input of the (total eight possible) Landolt C’s gap positions.

In the newly developed (DPMA, DRN 2019080916463800DE) method for measuring halo expansion (“Halometry,” see Figure 2), moving (8-orientation) Landolt Cs of VA level 0.0125 (corresponding to logMAR 1.9) were placed on the dark cylindrical projection surface of the simulator (radius 3.2 m). The luminance of the background was fixed at 0.032 cd/m^2^ (identical to the background luminance of clinical measurement methods for mesopic contrast vision without glare, such as in the Optovist I device used in this study, Vistec AG, Olching, Germany).

The contrast level of the Landolt Cs was 80% (Weber contrast). Like kinetic perimetry, Landolt Cs moved at a constant angular velocity of 1°/s – this velocity being a good compromise between spatial resolution and examination time for this examination situation (see Johnson and Keltner, 1987). All vectors originated from an identical origin (2.5° left and 0.6° below the centrally located green fixation mark), corresponding to the location of the static LED “array” glare source (left headlight of the oncoming car, constant illuminance level = 0.159 lux). Centrifugal vectors along the 0°, 45°, 90°, 135°, 180°, 225°, 270°, and 315° meridians defined stimulus motion (as highlighted by the blue arrows in Figure 2). The gap positions of the Landolt Cs were not allowed to point directly to the glare source or to the ±45° adjacent positions. Three to five gap positions were randomly presented per vector (3-of-5 criterion: at least three correct responses but no more than five trials). The onset of each visual signal was signaled by an auditory beep. The participant had the task of first pressing any key on the input device as soon as he or she thought to recognize the respective gap position of the Landolt C appearing from the non-sighted area. At this moment, the Landolt C was blanked and the local response time (“response time” = RT) was recorded, in milliseconds. In a second step, the participant’s task was to specify the gap location of the Landolt C, by pressing the corresponding position key. Halo extent was quantified in terms of the median of the vector lengths (i.e., eccentricity, in degrees visual angle) defined by the vector peaks.

## Results

Fifteen people participated: 5 females and 10 males, with a median age of 67.2 years (interquartile range = IQR 13.8, minimum 54.6, maximum 80.6).

The median binocular photopic high contrast decimal VA was 1.25 (0.5–2.0; IQR 0.6) and the median photopic low contrast binocular decimal low contrast VA was 0.41 (0.13–0.76; IQR 0.2).

The median clinically assessed binocular CS was log CS = 0.3 (0.1–0.7; IQR 0.4) without glare and log CS = 0.2 (0.1–0.7; IQR 0.3) with glare.

The median log (s) C-Quant parameter was 1.27 (0.93–1.52; IQR 0.29) for the leading eye and 1.23 (0.93 – 1.52; IQR 0.27) for the subdominant eye.

**FIGURE 3:**
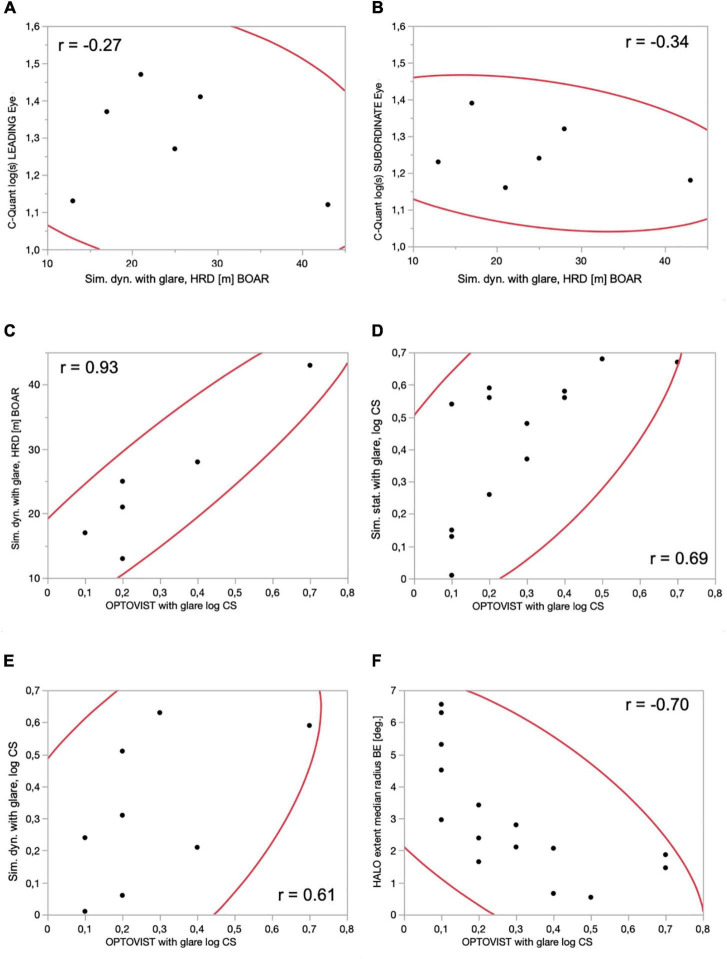
Correlations between various visual function parameters, with associated 95% confidence intervals (red lines) and correlation coefficients (text). **(A)** *Dynamic* conditions, hazard recognition distance (m) BOAR (Weber contrast 87%) vs. straylight perception: C-Quant, log (s), LEADING eye; **(B)** *dynamic* conditions, hazard recognition distance (m) BOAR (Weber contrast 87%) vs. straylight perception: C-Quant, log (s), SUBORDINATE eye; **(C)** contrast sensitivity: Optovist I WITH glare, log CS vs. *driving simulator, dynamic* conditions, hazard recognition distance (m) BOAR (Weber contrast 87%); **(D)** contrast sensitivity: Optovist I WITH glare, log CS vs. *driving simulator, static* conditions, ADAPTIVE thresholding algorithm (BestPEST), log CS; **(E)** contrast sensitivity: Optovist I WITH glare, log CS vs. *driving simulator, dynamic* conditions, ADAPTIVE thresholding algorithm (BestPEST), log CS; **(F)** contrast sensitivity: Optovist I WITH glare, log CS vs. HALO extent (radius in°), obtained with 8 position Landolt Cs (visual acuity level 0.0125, Weber contrast 80%), moving outward (1°/s) from a static, slightly eccentric LED glare source emanating from the center of a LED glare source, located 2.5° left and 0.6° below the centrally located green fixation mark.

Figure 3 shows correlations between key visual function parameters (for a full multivariate analysis of all correlations, see Supplementary Figure 1). The correlation between intraocular straylight perception, log (s), and HRD under glare conditions was poor for the 87% Weber contrast obstacle (leading/subdominant eye: *r* = −0.27/*r* = −0.34). Straylight perception exhibited an at most moderate correlation with those aspects of visual function measured in this study (CS, halo size, and HRD). The only exception was the negative correlations of log (s) of the *subordinate* eye with (binocular) log CS during glare exposition in the driving simulator, which was observed under both static and dynamic viewing conditions (see Supplementary Figure 1). Conversely, static log CS, measured under glare using a commercial clinical device (Optovist I), strongly predicted (*r* = 0.93) HRD under glare conditions for the 87% Weber contrast obstacle (see Figure 3). This was – to a lesser degree – also the case for obstacles with even lower contrast levels (see Supplementary Figure 1). Clinically assessed static log CS under glare conditions was also correlated with log CS measured within the driving simulator itself – under *static* (*r* = 0.69) as well as under *dynamic* (*r* = 0.61) conditions, and also with halo extent, measured with “halometry” (*r* = −0.70).

**FIGURE 4:**
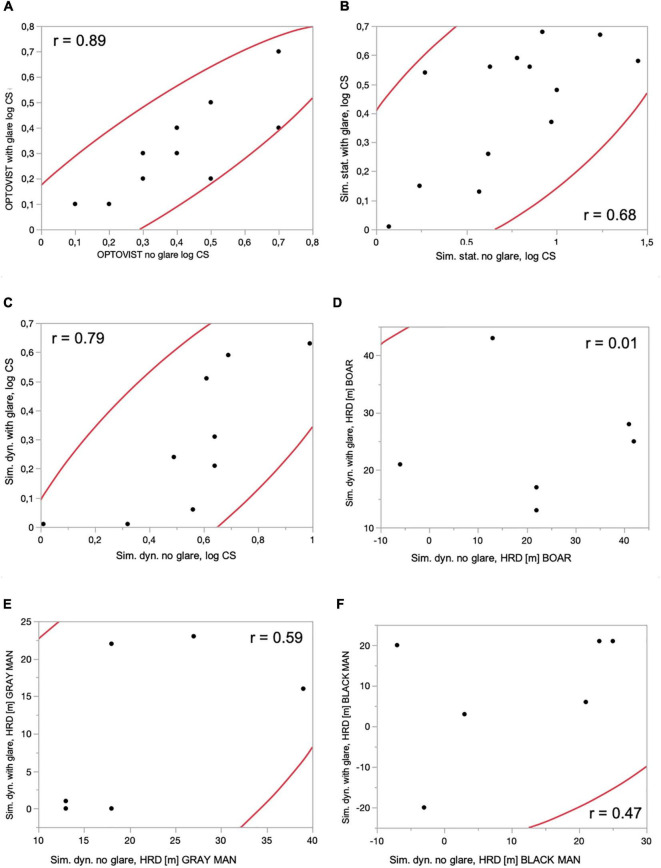
Correlations between log CS and hazard detection distances – when comparing the *with* and *without glare* conditions, under clinical (Optovist I) or driving simulator conditions, with associated 95% confidence ellipses and correlation coefficients. **(A)** *Clinical tests*: contrast sensitivity (log CS): Optovist I *without* glare and *with* glare. **(B)** *Driving simulator*: contrast sensitivity (log CS): *static* conditions, ADAPTIVE thresholding algorithm (BestPEST), *without* and *with* glare exposition. **(C)** *Driving simulator*: contrast sensitivity (log CS): *dynamic* conditions, ADAPTIVE thresholding algorithm (BestPEST), *without* and *with* glare exposition. **(D)** *Driving simulator*: *dynamic* conditions, hazard recognition distance (m) BOAR (Weber contrast 87%), *without* and *with* glare exposition. **(E)** *Driving simulator*: *dynamic* conditions, hazard recognition distance (m) GRAY MAN (Weber contrast 81%), *without* and *with* glare exposition. **(F)** *Driving simulator*: *dynamic* conditions, hazard recognition distance (m) BLACK MAN (Weber contrast 29%), *without* and *with* glare exposition.

As shown in Figure 4, log CS values with and without the presence of glare were closely correlated (*r* = 0.89), when measured under mesopic conditions using the clinical Optovist I. This was also the case for log CS measured in the driving simulator, when comparing log CS *with* and *without* glare both under *static* (*r* = 0.68) and dynamic conditions (*r* = 0.79). The correlations with regard to hazard detection distances under dynamic conditions within the driving simulator were lower, when comparing the *with* and *without* glare conditions for three different obstacle contrast levels (87% Weber contrast: *r* = 0.01; 81% Weber contrast: *r* = 0.59; and 29% Weber contrast: *r* = 0.47). Note that for brevity, we have focused here only on those correlations that were apparent in the data. For a full multivariate analysis and corresponding matrix containing all correlations of log CS and hazard detection distances – when comparing the *with* and *without glare* conditions, under clinical (Optovist I) or driving simulator conditions, see Supplementary Figure 2.

**FIGURE 5:**
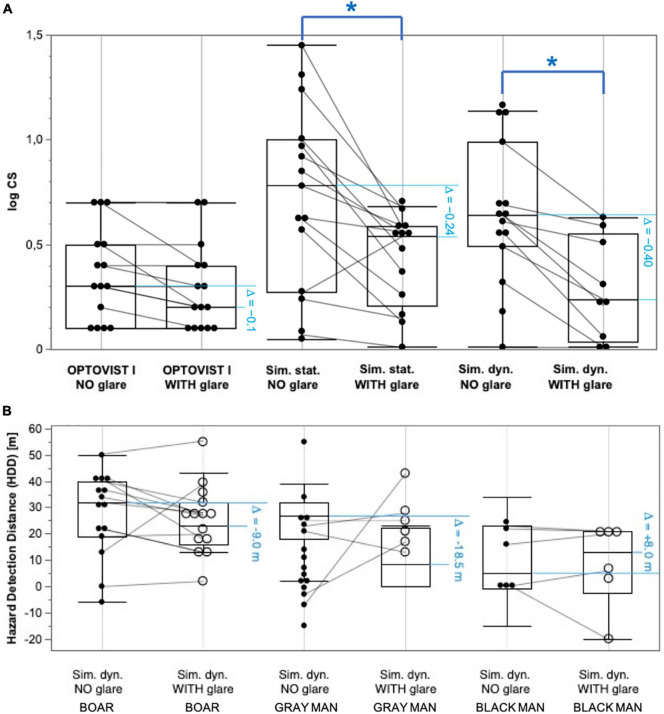
Pairwise comparisons (*without* vs. *with* glare) of log CS frequency distributions and hazard recognition distance (HRD) distributions, each shown as box and whisker plots: lower and upper limits of the box indicate the position of the 25th and 75th percentile, respectively, and thus represent the interquartile range (IQR); the enclosed horizontal line within the box visualizes the median. The whiskers symbolize the 1.5-fold of the IQR; in addition, the individual results are shown as black dots (local overlaps of dots are possible). Gray lines connect the results of the respective test person. Asterisks (*) mark statistically significant differences between *without* and *with* glare conditions. **(A)** *Clinical test*: log CS, obtained with Optovist I (*without* and *with* glare); *Simulator test*: *static* conditions, ADAPTIVE thresholding algorithm (BestPEST) *without* glare; *static* conditions, ADAPTIVE thresholding algorithm (BestPEST) *with* glare; *dynamic* conditions, ADAPTIVE thresholding algorithm (BestPEST) *without* glare; *dynamic* conditions, ADAPTIVE thresholding algorithm (BestPEST) *with* glare. **(B)** *Simulator test*; hazard recognition distance (HRD): *dynamic* conditions, hazard recognition distance (m) BOAR (Weber contrast 87%), *without* glare*; dynamic* conditions, hazard recognition distance (m) BOAR (Weber contrast 87%), *with* glare*; dynamic* conditions, hazard recognition distance (m) GRAY MAN (Weber contrast 81%), *without* glare; *dynamic* conditions, hazard recognition distance (m) GRAY MAN (Weber contrast 81%), *with* glare; *dynamic* conditions, hazard recognition distance (m) BLACK MAN (Weber contrast 29%), *without* glare; *dynamic* conditions, hazard recognition distance (m) BLACK MAN (Weber contrast 29%), *with* glare. The horizontal blue pairs of lines indicate the amount of change between the conditions of glare exposition and the related baseline condition (*without* glare).

Figure 5A shows the impact/reduction of glare on the median values of log CS, in relation to the baseline condition *without* glare. In case of clinical assessment of mesopic log CS with the Optovist I instrument, the reduction induced by glare was comparatively small (−0.1 log units); for the driving simulator setting the glare-induced log CS reduction increased to −0.24 log units, in case of *static* and to even −0.4 log units in case of *dynamic* conditions.

Referring to the median values of HRD s (see Figure 5B) in the driving simulator under dynamic conditions, glare exposition goes in hand with a deterioration (i.e., reduction) by −9.0 m (related to the baseline scenario *without* glare in case of an obstacle Weber contrast level of 87%), to −18.5 m (object Weber contrast level of 81%). For the (comparatively small) number of observations at the lowest obstacle Weber contrast level (29%) there was an increase (improvement) of HRD by 8.0 m.

Pairwise Wilcoxon tests for the comparison of conditions with and without glare showed the results presented in Table 2.

**TABLE 2:** Results of pairwise Wilcoxon tests for the comparison of conditions with and without glare.

Parameter compared with regard to conditions with and without glare	*p*-Value
Clinical assessment of mesopic log CS with the Optovist I device	*p* = 0.031
Assessment of log CS in the driving simulator, static condition	*p* = 0.003 (*)
Assessment of log CS in the driving simulator, dynamic condition	*p* = 0.008 (*)
Assessment of hazard recognition distances (level of log CS ≥ 0.06)	*p* > 0.999
Assessment of hazard recognition distances (level of log CS ≥ 0.09)	*p* = 0.094
Assessment of hazard recognition distances (level of log CS ≥ 0.54)	*p* = 0.625

Significant values with a Bonferroni-corrected critical *p*-value of 0.05/6 = 0.008 are marked with an asterisk (*).

## Discussion

This study implemented standardized testing of contrast vision/twilight vision, glare sensitivity, and halo extent, in a (nighttime) driving simulator setting. Each of these parameters was measured both under static and dynamic viewing conditions with and without glare, respectively.

### Comparison to previous studies

According to previous studies, both glare (Aulhorn and Harms, 1970) and ego-motion (Erkan et al., 2021) impair mesopic vision (i.e., VA and/or CS). Consistent with these, the present study showed a profound reduction of the CS under dynamic conditions *with* glare, compared to dynamic conditions *without* glare.

Clinical examinations using the Optovist I device showed a reduction of CS *with* glare, compared to the CS *without* glare to lesser extent. The reason for that could potentially be seen in a compensatory increase of the background luminance level during glare exposition in the Optovist I device (for measurements in the presence of glare, the background luminance is increased from 0.032 to 0.1 cd/m^2^) that do not take place in the driving simulator. Another reason could be due to the fact that the glare source in the driving simulator is more intense than in the Optovist I device.

Babizhayev (2003) stated that CS and glare sensitivity should be added to the regular requirements for a driver’s license, at least for older drivers. The present findings may be seen as consistent with this, in that they demonstrate an association of both recognition distance and halo extent with clinically obtained CS. However, recognition distance and halo extent may not be directly related to higher accident rates (for instance, the increase of these values may still be within safe breaking distances). Further studies are required to demonstrate the utility and efficacy of such an approach.

### Problems with regard to the specification of cut-off values

In the current study, various aspects of visual function were measured: VA, CS and straylight (see section “Methods”). In principle, various different cut-off criteria could be applied to these values in order to reasonably categorize people as either “fit” vs. “unfit” to drive. For instance, in Germany, fitness to drive is assumed for subjects with a VA of at least 0.4/0.2 (0.7 for both eyes in case of screening tests), and a CS of at least 1:23 (Aulhorn contrast, corresponding to a Weber contrast of 96%) (DOG and BVA, 2019). For C-Quant measurement, a cut-off criterion for driving of log (s) ≤ 1.5 was suggested (van den Berg et al., 2013).

Within the current study, none of the participants failed to meet the criteria with regard to clinical assessment of VA and CS, while for C-Quant measurements, one (two) participant/s failed with regard to the leading (subdominant) eye.

It is not yet known what the ideal way is to combine these parameters between the two eyes — or with other additional parameters like visual field loss — in order to accurately categorize people as fit or unfit to drive (i.e., in order to predict who is most likely to be involved in a motor accident).

### Simulation of glare

In the present study we used the nighttime driving simulator of the Aalen Mobility Perception and Exploration Lab (AMPEL) (see section “Methods” for more information on the setup) to realistically simulate the glare from oncoming car headlights. Other groups have used different approaches. For example, Hwang and Peli implemented a nighttime driving simulator in which glare was simulated via a programmable LED display board and a beamsplitter. The LED lights, representing headlights of oncoming cars, can be superimposed on the driving simulator screen (Hwang and Peli, 2013). In comparison to the AMPEL glare system implemented in the present study, the approach by Hwang and Peli leads to reflections (“ghosting”) of the glare sources that are unrealistic, and may be seen as liable to distract test subjects. Though, in the interest of balance, it should be noted that during nighttime driving, small distractions may be overshadowed by the headlight glare of the oncoming car, and there is no directly evidence that such small distractions bias experimental results on headlight glare studies.

Another option for the induction of glare during nighttime driving simulation was expounded by Haycock et al. (2017) who developed a hybrid display system that consisted of usual projectors as well as LED panels mounted on a robotic actuator. However, unlike the AMPEL simulator, the LED panels remained visible even when LEDs are not turned on, which could disturb the tested subjects.

The Fraunhofer IAO developed a method for measuring the physiological glare caused by oncoming vehicles in a driving simulator using pairs of numbers of different contrast (Melcher et al., 2011). This approach used glare inducing LEDs that were mounted in the plane of the front windshield which again might affect the realism of this experimental setup as the distance of the LEDs does not seem to be realistic. The stimuli used for determining contrast vision (by showing two-digit numbers of different contrasts at different times in relation to the time of glare and measuring reaction and error rate) in these experiments were also potentially problematic, in that they consisted of numerical optotypes, and VA requirements are known to differ markedly between digits (e.g., Wesemann et al., 2010).

In short, the present authors consider the AMPEL driving simulator used in this study to be more realistic and immersive than those solutions published previously. However, a driving simulator is always limited compared to real, on-road experiments (which, in turn, have their own weaknesses and should therefore be complemented by simulator studies). In real road traffic, numerous distractions and unpredictable events can occur, and the dangers are much greater.

### Study limitations

Due to COVID, only a comparatively small sample size could be examined, with corresponding restrictions of representativity. In particular, visual function was comparatively good amongst our subjects, so the effects of advanced opacities of the refractive media are not covered by the present study. All tested subjects belong to the middle-aged or to the advanced age group (55–80 years). No conclusions can therefore be drawn for younger subjects.

All VA measurements (at high and low contrast levels) were carried out under photopic conditions. In future studies, low contrast VA measurements should be also assessed under mesopic conditions – in accordance with the nighttime driving simulator setting in order to make results more comparable.

In this study, data analysis was focused on hazard detection, as it was assumed that the difference between the recorded detection and recognition distance was mainly attributable to the time span required to verbalize the response. However, detection and recognition/discrimination are different visual tasks, therefore future studies to examine detection distance and recognition/discrimination distance separately would be of interest.

In addition, this article covers glare disability, but not glare recovery. Thus, once retinal photopigment is bleached by an intense light source, it takes time for normal visual function to recover. During the time, drivers are limited in their visual performance and may not be able to assess hazards correctly. This may affect, for example, a driver’s ability to perceive hazards immediately upon exiting a dark tunnel, or when transitioning from dark countryside into a bright urban environment. Glare recovery times, specifically for low-contrast stimuli, are known to increase with age (Schieber, 1994). Glare recovery speed may thus be an extremely important additional factor to consider – particularly regarding night-driving ability in older drivers – and this added temporal component should be considered in future studies.

Conditions for clinical and driving simulator examinations were not exactly comparable for different reasons. This may have influenced correlations between clinical and simulator measures: For instance, the Optovist I device has *one* single static glare source with a color temperature of 7,800 K in front of a background with a luminance of 0.1 cd/m^2^ (with glare) or 0.032 cd/m^2^ (without glare), whereas a VW Rabbit VII has *two* headlights with a color temperature of 3,000 K. The apron luminance of a VW Rabbit VII at a distance of 10–40 m is 0.2–2.0 cd/m^2^. Comparing the glare source in the Optovist I with that of a VW Rabbit VII, the glare source in the Optovist I corresponds to that of a Rabbit headlight (low beam) in terms of luminance at a distance of 30 m, in terms of vision angle at a distance of 50 m and in terms of eccentricity at a distance of 23 m.

Finally, it is important to note that a long-term longitudinal study would be required to establish how well the basic parameters of visual function, such as CS or halo extent, examined in the present study, predict long-term driving habits and accident rates. From the authors’ perspective such a future validation should take the form of a prospective, long-term (data protection-compliant) video monitoring of driving habits and (near) crash events in the test participants’ own vehicles (Huisingh et al., 2019), instead of aiming at short-term method-equivalent on-road tests. In this way, it could be examined how parameters investigated in this study, such as HRD in the simulator correspond to (near) crash involvement on-road, or whether there is a relation between CS measured in the simulator under motion and on-road driving habits.

## Conclusion

In conclusion, intraocular straylight (C-Quant) was not strongly associated with CS or other vision-related variables. Conversely, CS as measured in the simulator was strongly associated with HRD, halo extent and clinically obtained CS (Optovist I instrument). The presence of glare and motion lead to a further degradation/deterioration of CS in a well-standardized driving simulator environment. Future prospective studies are necessary to validate CS, intraocular straylight, and halo extent for predicting fitness to drive/driving ability under real-life conditions.

## Data availability statement

The raw data supporting the conclusions of this article will be made available by the authors, without undue reservation.

## Ethics statement

The studies involving human participants were reviewed and approved by the Institutional Review Board of the State Medical Association of Baden-Württemberg (F-2015-044#A2). The patients/participants provided their written informed consent to participate in this study.

## Author contributions

JU, US, PE, and MW contributed substantially to the conception and design of the work and to the acquisition of the data. JU, US, MW, DC, and PJ contributed substantially to the analysis and interpretation of the data, the drafting of the work, and the critical revision. All authors provided approval for publication of the content and agreed to be accountable for all aspects of the work.

## Supplementary material

Supplementary Figure 1 Visualization of the multivariate analysis of visual function parameters, mainly under glare exposition, under clinical (C-Quant, Optovist I) or driving simulator in the form of a matrix with associated 95% confidence ellipses and correlation coefficients. The measured variables are plotted on the main diagonal from top left to bottom right. The following variables are shown (descending from top left; all binocular with the exception of C-Quant data): *Clinical tests*: straylight perception: C-Quant, log (s), LEADING eye; C-Quant log (s), SUBORDINATE eye; contrast sensitivity: Optovist I WITH glare, log CS. *Driving simulator*: contrast sensitivity (log CS), WITH glare exposition, each: *task #6*: *static* conditions, ADAPTIVE thresholding algorithm (BestPEST); *task #7*: *dynamic* conditions, ADAPTIVE thresholding algorithm (BestPEST); *task #8.1*: *dynamic* conditions, hazard recognition distance (m) BOAR (Weber contrast 87%); *task #8.2*: *dynamic* conditions, hazard recognition distance (m) GRAY MAN (Weber contrast 81%); *task #8.3*: *dynamic* conditions, hazard recognition distance (m) BLACK MAN (Weber contrast 29%). HALO extent (radius in°), obtained with 8 position Landolt Cs (visual acuity level 0.0125, Weber contrast 80%), moving outward (1°/s) from a static, slightly eccentric LED glare source emanating from the center of a LED glare source, located 2.5° left and 0.6° below the centrally located green fixation mark.

Supplementary Figure 2 Visualization of the multivariate analysis of log CS and hazard detection distances – when comparing the *with* and *without glare* conditions, under clinical (Optovist I) or driving simulator conditions in the form of a matrix with associated 95% confidence ellipses and correlation coefficients. The measured variables are plotted on the main diagonal from top left to bottom right. The following variables are shown (descending from top left; all binocular with the exception of C-Quant data): *Clinical tests*: Contrast sensitivity (log CS): Optovist I *without* glare and *with* glare. *Driving simulator*: Contrast sensitivity (log CS): *task #2*: *static* conditions, ADAPTIVE thresholding algorithm (BestPEST), *without* glare exposition; *task #3*: *dynamic* conditions, ADAPTIVE thresholding algorithm (BestPEST), *without* glare exposition; *task #4.1*: *dynamic* conditions, hazard recognition distance (m) BOAR (Weber contrast 87%), *without* glare exposition; *task #4.2*: *dynamic* conditions, hazard recognition distance (m) GRAY MAN (Weber contrast 81%), *without* glare exposition; *task #4.3*: *dynamic* conditions, hazard recognition distance (m) BLACK MAN (Weber contrast 29%), *without* glare exposition; *task #6*: *static* conditions, ADAPTIVE thresholding algorithm (BestPEST), *with* glare exposition; *task #7*: *dynamic* conditions, ADAPTIVE thresholding algorithm (BestPEST), *with* glare exposition; *task #8.1*: *dynamic* conditions, hazard recognition distance (m) BOAR (Weber contrast 87%), *with* glare exposition; *task #8.2*: *dynamic* conditions, hazard recognition distance (m) GRAY MAN (Weber contrast 81%), *with* glare exposition; *task #8.3*: *dynamic* conditions, hazard recognition distance (m) BLACK MAN (Weber contrast 29%), *with* glare exposition. Corresponding variables *with* and *without* glare are marked by blue squares.
