# Supplementary material for: Does intraocular straylight predict night driving visual performance? Correlations between straylight levels and contrast sensitivity, halo size, and hazard recognition distance with and without glare
Source: Front Hum Neurosci. 2022 Sep 13;16:910620. doi: 10.3389/fnhum.2022.910620 (PMC9514855; doi:10.3389/fnhum.2022.910620)
Supplement: Supplementary file 1 [file Data_Sheet_1.DOCX]

**Additional information on the calibration of the headlamps**

In order to calibrate the LED glare sources, the luminance values (measured with the calibrated spectroradiometer CAS 140 VIS/UV, Instrument Systems GmbH, Munich, Germany) of low-beam headlamps of the real-world glare vehicle (VW Rabbit/Golf Type VII) have been measured in the field under typical night driving conditions at distances between 10 m and 100 m.

For the calibration measurements, the real-world glare vehicle has been oriented parallel to the lane with an accuracy of about ± 5°. For all distances the spectrometer has been positioned on the adjacent lane, the horizontal offset to the center of the left headlamp was 1.2 meters. In the vertical direction the entrance of the spectrometer head has been positioned at a height of 1.2 meters above street level, representing the eye height of a typical driver.

During the real-world test drives the supervising persons paid close attention to proper lane keeping of the driver.

During the simulator runs the left and right glare sources were commanded to the luminance (and diameter) settings corresponding to the simulated distance as indicated by the real-world measurements. Thus the luminance of the glare sources corresponds to the luminance of the real-world glare source. Consequently, the measured effect of beam directionality during the passing by of the real-world headlamps could fully be incorporated in the simulator experiments.

The determination of the spatial distribution of apron luminance in the absence of glare sources has been performed in order to get the baseline for the proper simulation of the effect of own vehicles headlamps. For these measurements, a digital camera Canon EOS 600D (Canon Inc., Tokyo, Japan) was used. The dynamic range of apron luminance (without glare sources) was well within the 8 bit dynamic range of the digital camera. The dynamic range of measurements performed via digital cameras can easily be enhanced by the utilization of different f-stops, ISO settings and integration times, as long as saturated image details are not evaluated. For the spatial distribution of apron luminance in the absence of glare sources this has not been necessary.
