# Supplementary figures and images for: Does intraocular straylight predict night driving visual performance? Correlations between straylight levels and contrast sensitivity, halo size, and hazard recognition distance with and without glare
Source: Front Hum Neurosci. 2022 Sep 13;16:910620. doi: 10.3389/fnhum.2022.910620 (PMC9514855; doi:10.3389/fnhum.2022.910620)

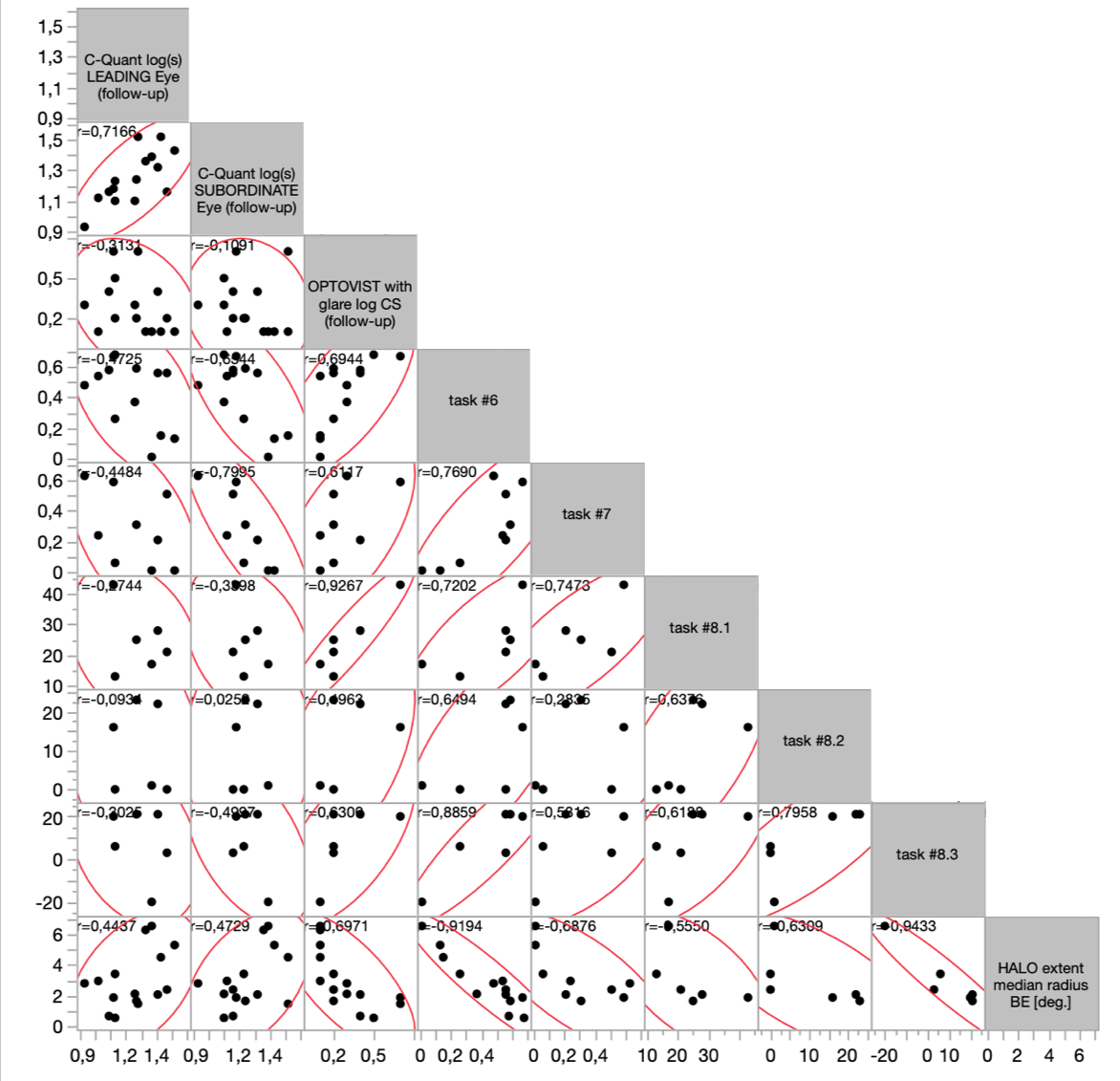

Supplement: Supplementary Figure 1 — Visualization of the multivariate analysis of visual function parameters, mainly under glare exposition, under clinical (C-Quant, Optovist I) or driving simulator in the form of a matrix with associated 95% confidence ellipses and correlation coefficients. The measured variables are plotted on the main diagonal from top left to bottom right. The following variables are shown (descending from top left; all binocular with the exception of C-Quant data): Clinical tests: straylight perception: C-Quant, log (s), LEADING eye; C-Quant log (s), SUBORDINATE eye; contrast sensitivity: Optovist I WITH glare, log CS. Driving simulator: contrast sensitivity (log CS), WITH glare exposition, each: task #6: static conditions, ADAPTIVE thresholding algorithm (BestPEST); task #7: dynamic conditions, ADAPTIVE thresholding algorithm (BestPEST); task #8.1: dynamic conditions, hazard recognition distance (m) BOAR (Weber contrast 87%); task #8.2: dynamic conditions, hazard recognition distance (m) GRAY MAN (Weber contrast 81%); task #8.3: dynamic conditions, hazard recognition distance (m) BLACK MAN (Weber contrast 29%). HALO extent (radius in°), obtained with 8 position Landolt Cs (visual acuity level 0.0125, Weber contrast 80%), moving outward (1°/s) from a static, slightly eccentric LED glare source emanating from the center of a LED glare source, located 2.5° left and 0.6° below the centrally located green fixation mark. [file Image_1.TIFF]

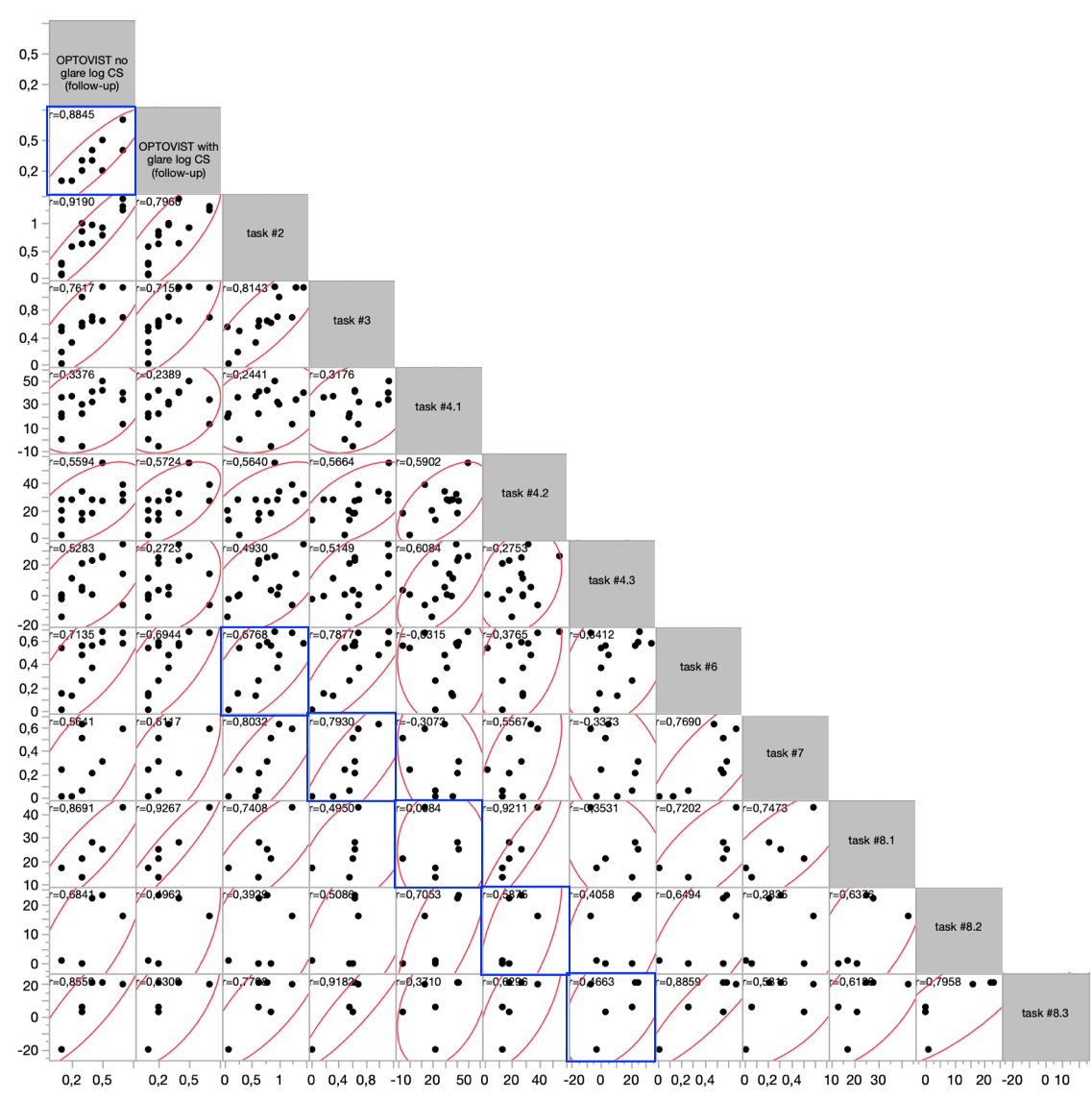

Supplement: Supplementary Figure 2 — Visualization of the multivariate analysis of log CS and hazard detection distances – when comparing the with and without glare conditions, under clinical (Optovist I) or driving simulator conditions in the form of a matrix with associated 95% confidence ellipses and correlation coefficients. The measured variables are plotted on the main diagonal from top left to bottom right. The following variables are shown (descending from top left; all binocular with the exception of C-Quant data): Clinical tests: Contrast sensitivity (log CS): Optovist I without glare and with glare. Driving simulator: Contrast sensitivity (log CS): task #2: static conditions, ADAPTIVE thresholding algorithm (BestPEST), without glare exposition; task #3: dynamic conditions, ADAPTIVE thresholding algorithm (BestPEST), without glare exposition; task #4.1: dynamic conditions, hazard recognition distance (m) BOAR (Weber contrast 87%), without glare exposition; task #4.2: dynamic conditions, hazard recognition distance (m) GRAY MAN (Weber contrast 81%), without glare exposition; task #4.3: dynamic conditions, hazard recognition distance (m) BLACK MAN (Weber contrast 29%), without glare exposition; task #6: static conditions, ADAPTIVE thresholding algorithm (BestPEST), with glare exposition; task #7: dynamic conditions, ADAPTIVE thresholding algorithm (BestPEST), with glare exposition; task #8.1: dynamic conditions, hazard recognition distance (m) BOAR (Weber contrast 87%), with glare exposition; task #8.2: dynamic conditions, hazard recognition distance (m) GRAY MAN (Weber contrast 81%), with glare exposition; task #8.3: dynamic conditions, hazard recognition distance (m) BLACK MAN (Weber contrast 29%), with glare exposition. Corresponding variables with and without glare are marked by blue squares. [file Image_2.TIFF]
